# Comparing the psychosocial impacts of COVID-19 in seven low- and middle-income countries: A cross-sectional study

**DOI:** 10.1371/journal.pgph.0005944

**Published:** 2026-06-16

**Authors:** Sandila Tanveer, Philip J. Schluter, Richard J. Porter, Joseph M. Boden, Ben Beaglehole, Ruqayya Sulaiman-Hill, Shaystah Dean, Wafaa N. Al-Hussainni, Muthana A. Majid Al-Masoodi, Maliheh Arshi, Jacob A. Bentley, Mehmet Dinç, Norah Feeny, Hamse Jibriil Ibrahim, Ahmed Muse Ismail, Ahmed Said Ismail, Mussarat Jabeen Khan, Mohammad Sabzi Khoshnami, Mohamed Ahmed Kunle, Daniella Levine, Amir Moghanibashi-Mansourieh, Aaron Moratz, Amer Siddiq Amer Nordin, Sara Noruzi, Saadet Öztürk, Anggi Rahajeng, Shaista Shaikh, Nisa Tanveer, Feyza Topçu, Irfan Yunianto, Lori A. Zoellner, Caroline Bell

**Affiliations:** 1 Department of Psychological Medicine, University of Otago, Christchurch, New Zealand; 2 Te Kaupeka Oranga, Faculty of Health, Te Whare Wānanga o Waitaha, University of Canterbury, Christchurch, New Zealand; 3 School of Clinical Medicine, Primary Care Clinical Unit, University of Queensland, Brisbane, Australia; 4 Department of Psychological Medicine, University of Otago, Wellington, New Zealand; 5 Basic Sciences Department, Ibn Sina University of Medical and Pharmaceutical Sciences, Baghdad, Iraq; 6 Department of Scholarships and Cultural Relations, Mustansiriyah University, Baghdad, Iraq; 7 Department of Social Work, University of Social Welfare and Rehabilitation Sciences, Tehran, Iran; 8 Department of Rehabilitation Medicine, University of Washington, Seattle, Washington, United State of America; 9 Department of Psychology, Hasan Kalyoncu University, Gaziantep, Turkiye; 10 College of Arts and Sciences, Case Western Reserve University, Ohio, United State of America; 11 Student Relations, University of Burao, Burao, Somaliland; 12 Department of Advanced Studies, University of Burao, Burao, Somaliland; 13 Animal Science and Agriculture, University of Burao, Burao, Somaliland; 14 Department of Psychology, International Islamic University, Islamabad, Pakistan; 15 Research and Community Service, University of Burao, Burao, Somaliland; 16 Postgraduate Studies, University of Burao, Burao, Somaliland; 17 Department of Psychological Medicine, Universiti Malaya, Kuala Lumpur, Malaysia; 18 Lorestan University of Medical Sciences, Khorramabad, Iran; 19 Department of Economics and Business, Vocational College, Universitas Gadjah Mada, Yogyakarta, Indonesia; 20 Department of Psychology, Islamabad Model College for Girls (Postgraduate), Islamabad, Pakistan; 21 Department of Peace and Conflict Sciences, National Defence University, Islamabad, Pakistan; 22 Faculty of Teacher Training and Education, Universitas Ahmad Dahlan, Yogyakarta, Indonesia; 23 Department of Psychology, University of Washington, Seattle, Washington, United State of America; Universitas Muhammadiyah Aceh, INDONESIA

## Abstract

The COVID-19 pandemic inflicted widespread and prolonged stress, particularly in low- and middle-income countries (LMICs). Similar events are likely in the future, and understanding moderators of the effects in LMICs may be helpful. An observational study of adults aged ≥18 years in Türkiye, Indonesia, Iraq, Malaysia, Pakistan, Somaliland, and Iran was conducted using an online survey between 2021 and 2023. Data were collected on sociodemographic and pandemic-related variables along with standardized measures of psychological distress (Kessler Psychological Distress Scale 10, K10; Posttraumatic Stress Disorder Checklist for DSM-5, PCL5), wellbeing (World Health Organization-Five Well-Being Index, WHO-5), post-traumatic growth (Posttraumatic Growth Inventory, PTGI), and pandemic-related distress (COVID-19 Psychosocial Impacts Scale, CPIS). Modified Poisson multilevel mixed-effect models were used to explore associations. Cumulative distribution function plots were used to visually compare sites, followed by ANCOVA to assess statistical differences while controlling for covariates. Overall, 2,574 adults aged 18–73 years (M = 28.0 years, SD = 10.6) participated. They were predominantly female (64.6%), single (58.0%), and Muslim (92.2%), with 81.2% having at least a tertiary qualification. The CPIS score range was 5–144 (M = 47.7, SD = 24.0). Psychological outcomes revealed substantial distress on K10 (32.2%), PCL5 (37.8%), and WHO-5 (45.0%) measures. Being female, married, studying full-time, and unemployed were consistently linked to higher psychological distress. The PTGI scores ranged from 0-105 (M = 53.3, SD = 23.3), showing a wide spectrum of posttraumatic growth in responses to COVID-19. Country-wise comparisons indicated variations in distress, well-being, and post-traumatic growth in response to COVID-19. The variation in distress scores appeared to be influenced by individual (e.g., gender, marital status, education, employment) and contextual (e.g., lockdown) factors. These findings suggest that strategies aimed at mitigating long-term psychosocial impacts of pandemics in resource-constrained settings may have population-health beneficial. **Registration details:** The study is registered with the Clinical Trial Registry (NCT05052333).

## Introduction

The novel coronavirus disease 2019 (COVID-19) pandemic, declared a global health emergency by the World Health Organization (WHO) in March 2020 [1], triggered unprecedented public health measures and socioeconomic disruptions worldwide. Multiple waves of infections, driven by evolving variants [2], challenged global health systems and societies through 2023. The psychosocial consequences of the pandemic were profound [3], encompassing both negative and positive outcomes. Negative outcomes include anxiety, depression, post-traumatic stress disorder (PTSD), and substance use disorders [4,5]. Positive impacts, include surviving (coping well, meeting basic needs, and maintaining health) and thriving (self-development, reflection, and growth) [6,7]. Although the WHO declared the end of COVID-19 as a global health emergency in May 2023 [8], its psychosocial impacts continue to reverberate, particularly in low- and middle-income countries (LMICs) where resource constraints amplify vulnerabilities [9]. Studying these impacts remains critical to inform recovery strategies, strengthen mental health systems, and prepare for future pandemics, especially in under-resourced settings where psychosocial research is scarce.

LMICs face unique challenges that exacerbate the psychosocial toll of the pandemic. Limited healthcare infrastructure, economic instability, and entrenched social inequalities have intensified mental health burdens and hindered access to care [9–11]. Existing multinational studies on COVID-19’s psychosocial impacts in LMICs have primarily focused on negative outcomes, reporting elevated prevalence of psychological distress [4,12,13], anxiety [4,14], depression [4,5,12–14], and PTSD [12]. These studies employing standardized measures have identified risk factors such as younger age, female gender, and high COVID-19 exposure as predictors of depression [5] and stress [15] from non-representative samples. These studies have generally not considered positive psychosocial impacts, such as resilience or post-traumatic growth, which are critical for understanding holistic recovery. Post-traumatic growth refers to positive psychological changes that emerge following exposure to traumatic or challenging experiences [16], such as the COVID-19 pandemic. As LMICs transition to recovery, understanding the full spectrum of psychosocial impacts—both adverse and adaptive—can inform targeted interventions and policy reforms.

By examining seven LMICs—Indonesia, Iran, Iraq, Malaysia, Pakistan, Somaliland, and Türkiye—this study addresses these gaps by employing standardized measures of psychological distress, wellbeing, and post-traumatic growth, alongside the novel COVID Psychosocial Impacts Scale (CPIS), to capture both negative and positive impacts [17]. Unlike prior multinational studies, which focus on mental health disorders alone, this study examines the broader personal, social, and economic consequences of the pandemic while exploring positive adaptations in a cross-cultural context for the first time. By highlighting both challenges and opportunities, it contributes to global health equity and informs strategies to mitigate the long-term effects of pandemics in resource-constrained settings.

Conceptually, we posit that pandemic-related exposures influence psychosocial outcomes in both negative (e.g., distress, anxiety, depression, PTSD) and positive (e.g., post-traumatic growth) directions [18]. These relationships are shaped by sociodemographic and contextual factors, including age, gender, and country-specific vulnerabilities, particularly in LMICs with constrained healthcare, social, and economic resources [5,9–11,15]. In our study, we examine psychological distress and post-traumatic growth using both descriptive and analytical approaches, estimating associations between pandemic-related exposures and psychosocial outcomes while controlling for these sociodemographic and contextual variables. This framework allows us to capture both population-level patterns and country-specific variations, providing an understanding of the pandemic’s psychosocial impacts across diverse cultural settings.

## Methods

### Ethics statement

Ethical approval was granted by the Human Ethics Committee, University of Otago, New Zealand (Ref No. 21/102). In addition, collaborators obtained local authorisation or ethical approval in their respective host universities [Indonesia (Universitas Gadjah Mada Ethics Committee Ref. No. KE/UGM/008/EC/2021); Iraq (Ibn Sina University of Medical and Pharmaceutical Sciences, Institutional Review Board (IRB) - Ethical Committee); Malaysia (Universiti Malaya Research Ethics Committee, Ref. No. UM.TNC 2/UMREC); Pakistan (International Islamic University Islamabad, Institutional Ethical Review Committee, Ref. No. IIUI/ORIC/Ethical-certificate/2021); Somaliland (Washington Human Subjects Division (HSD) IRB ID: STUDY00015135); and Türkiye (Hasan Kalyoncu Üniversitesi Etik Ethics Committee Ref. No. E-97105791-050.01.01-2342)].

### Design

This is a multinational, non-randomized cross-sectional observational study examining the psychosocial impacts of the COVID-19 pandemic across seven LMICs.

### Setting and target population

Indonesia, Iran, Iraq, Malaysia, Pakistan, Somaliland, and Türkiye were purposefully and pragmatically selected, driven by our desire to capture global LMIC geographic diversity within a constrained budget across a variety of demographics, health systems, policies, and COVID-19 burdens and responses. Additionally, country-specific lead investigators required identification and consent to provide contextual insight, ensuring the survey was culturally appropriate, and assisting in data collection. This study forms part of our ongoing programme of research on events which impacted many immigrants from these countries [19].

### Measures

Data on sociodemographic and study variables were collected through an online survey, available in local languages and English (See S1 File).

#### Sociodemographic measures.

Sociodemographic measures include age, gender, marital status, religion, highest level of education, study or work status, and income status. Measures were tailored for each country to ensure contextual appropriateness, as advised by collaborators. For example, in the study or work status, the part-time study option was removed from the survey administered in Türkiye and the stay-home parent option was removed from the survey administered in Malaysia. In addition, in Iran, the item related to religion was modified by adding Zoroastrianism and Judaism (instead of Hinduism and Buddhism) to reflect the demographic profile of the region.

#### Prior trauma exposure.

Participants were asked to select as many as appropriate from a list of previous trauma exposure, including natural disasters, war or military conflict, childhood adversity, physical or sexual assault, and serious physical accident(s).

#### Pandemic-related variables.

Data was obtained on COVID-19 morbidity and mortality in the household, ongoing COVID-19 infection and lockdown at the time of data collection, COVID-19 vaccination status and intention, exposure to COVID-related information, and pandemic-related financial variables (job loss, essential worker).

#### COVID Psychosocial Impacts Scale (CPIS).

The 32-item CPIS was devised [20] using the ‘group mind’ process [21], which asked colleagues, including collaborators, to review and rigorously critique a draft of the questionnaire, with iterative improvements made based on their comments. The CPIS examined the psychosocial impacts of COVID-19. Of these, 28 items examine exposure to and amount of stress on a 5-point scale (1 = ‘yes, no stress at all’ to 5 = ‘yes, a lot of stress’) experienced in response to the pandemic-related life event. These events include personal and family impacts, changes to daily routines and behaviours, relationships, employment, income, and exposure to information about COVID-19. The remaining four items assess on a 5-point scale the perception of stress (1 = ‘Considerably less stressed’ to 5 = ‘Considerably more stressed’) and psychological wellbeing (1 = ‘Much better’ to 5 = ‘Much worse’), comparing overall life stress pre and post COVID-19. The total CPIS score ranges from 0-160, with higher scores reflecting higher pandemic-related stress. The scale demonstrated strong overall reliability scores (α = 0.906); additionally, none of the items were deleted from the scale.

#### Kessler Psychological Distress Scale-10 (K10).

This is a 10-item global measure of distress with items based on symptoms of anxiety and depression in the previous 30 days. Respondents rate each item on a 5-point rating scale (1 = ‘none of the time’ to 5 = ‘all of the time’) [22]. The K10 score ranges from 10-50, and a cut-off score of ≥30 is indicative of severe mental distress [23].

#### WHO Well-Being Index (WHO-5).

This is a 5-item measure assessing subjective psychological wellbeing in the previous two weeks [24]. It contains positively phrased items, with respondents rating each item on a 6-point rating scale (0 = ‘at no time’ to 5 = ‘all of the time’) of how they have felt over the past two weeks. The WHO-5 score ranges from 0-25, which can be multiplied by 4 to give a final score; with 100 representing the highest subjective wellbeing. A cut-off score of ≤50 is generally used to screen for clinical depression [24].

#### Post Traumatic Stress Disorder (PTSD) Checklist for DSM-5 (PCL-5).

This is a 20-item measure assessing symptoms of PTSD in the past month, anchored in response to COVID-19 as a potentially traumatic event. Respondents rate items on a 5-point rating scale (0 = ‘not at all’ to 4 = ‘extremely’). The PCL-5 score ranges from 0-80 [25], and a cut-off score of ≥33 is indicative of probable PTSD [26].

#### Posttraumatic Growth Inventory (PTGI).

This is a 21-item measure assessing post-traumatic growth and self-improvement [27], anchored in response to COVID-19 as the potentially traumatic event. There are five factors, such as relating to others (item no. 4, 10, 12, 19), new possibilities (item no. 3, 7, 11, 14, 17), improved relationships (item no. 6, 8, 9, 15, 16, 20, 21), spiritual growth (item no. 5, 18), and appreciation for life (item no. 1, 2, 13). Respondents rate items on a 6-point rating scale (0 = ‘not at all’ to 5 = ‘a lot’). The PTGI scores range from 0-105, with higher scores reflecting positive transformation after the traumatic event.

### Patient and public involvement

Feedback has been taken on the project design from academics in the countries of interest. Additionally, a pilot study was conducted with a small group of participants to assess language appropriateness and the practical administration of the survey, including clarity of translated items, survey length, and functionality across devices. Feedback from the pilot informed minor refinements to wording and survey flow to improve comprehension and administration. The pilot was not conducted for validation purposes, and pilot data were not included in the analyses; validation of the measures was conducted using data from the main study.

### Data collection and recruitment

Data were collected over a three-week period in each country to capture a specific set of circumstances at a single point in time. This was carried out during the transitional period marked by the emergence of the Omicron variant of COVID-19 in 2021 [2] and the subsequent winding down of the global emergency declaration in 2023 [8]. Due to varying timelines for establishing local collaborations and obtaining ethical approvals, data were collected in 2021 from Türkiye (20/12/2021–10/01/2022); in 2022 from Indonesia (21/01/2022–11/02/2022), Iraq (21/04/2022–12/05/2022), Malaysia (09/04/2022–30/04/2022), Pakistan (10/05/2022–31/05/2022), and Somaliland (30/10/2022–20/11/2022); and in 2023 from Iran (27/07/2023–17/08/2023), with each data collection period remaining open for three weeks.

The mode of recruitment varied between countries, but it generally involved the distribution of the anonymous survey link using departmental mailing lists or social media, led by country-specific collaborators at each host site. The researchers implemented snowball sampling techniques, where initial participants were recruited and encouraged to share the survey link with prospective participants, allowing the sample to grow progressively through referrals from those initial participants.

### Data management

To ensure the anonymity of the obtained dataset, the following standard operating procedures were implemented. Where applicable, only the collaborators hold contact information for their participant pool. Each site has specific guidelines to preserve anonymity specified in its local ethics application. Following collection, data was saved on the University of Otago, New Zealand, Qualtrics platform using an auto-generated non-identifiable letter-number string. Importantly, the obtained dataset was completely anonymous as no identifiable information was obtained in the survey.

### Sample size

Reliable population-level estimates for the primary outcomes were unavailable across the participating LMICs at the time of study design, therefore formal power calculations were not conducted. A target sample size of 500 participants per country was pragmatically selected, accounting for the descriptive and exploratory nature of this study, as well as feasibility considerations, comparability with similar multinational surveys conducted during the pandemic, and the need to minimize institutional burden [28]. Moreover, a target sample size of around 500 participants per country provides good power (≈80% or higher) to detect small-to-moderate effects in most common analyses, allows detection of meaningful heterogeneity in effect sizes across countries, and strikes a balance between cost and feasibility against statistical adequacy.

### Data analysis

Incomplete survey responses, defined as those with missing data on psychological outcomes, were removed from the analyses. Data were analysed descriptively and analytically using SPSS v29 [29] and Stata/SE 18.0 [30]. Spearman correlation coefficients (r_s_) were used to assess relationships between psychological outcomes (K10, PCL-5, WHO-5). Correlation strength was interpreted using the following thresholds: r_S_ values of 0.10–0.29 were considered weak, 0.30–0.69 moderate, and ≥0.70 strong [31].

To relate exposures to outcomes, modified Poisson regression in multilevel mixed-effects modelling [32] was used for a binary outcome with non-rare prevalence, accounting for the data's hierarchical structure and estimating relative risks. The model incorporated both fixed and random effects, with countries as the clustering variable and time as a random coefficient to capture country-specific temporal variations. This approach enabled estimation of population-averaged effects and country-specific deviations in temporal trends while adjusting for within-country correlation. Robust standard errors were used to address potential model misspecification. Model diagnostic checks centred around evaluation of likelihood ratio tests comparing hierarchies, and the identification of any convergence issues or parameter instability. Poor convergence or parameter instability signals overly complex random‑effects structures; weakly identified variance components; and, quasi‑separation or sparse data. Furthermore, variance components and intraclass correlation coefficients, which give the degree of clustering explained by higher‑level units, were checked. While undertaken, these evaluations were not explicitly reported here, unless found to be either problematic or important.

To examine empirical distributions of standardized psychological measures across countries, superimposed cumulative distribution function (CDF) plots were created, displaying cumulative percentages with an interpolation line to highlight differences. Subsequently, one-way ANOVA was conducted to assess statistical significance, followed by effect size estimation to quantify the magnitude of differences. To control for the potential effects of age and education on differences between countries, an ANCOVA was conducted. Age can influence life experience, coping strategies, and psychological resilience, while education reflects socioeconomic and cognitive resources that may affect wellbeing and survey responses. Controlling for these factors was undertaken to reduce confounding and allow a clearer assessment of the primary variables of interest.

Reporting followed ‘Strengthening the Reporting of Observational Studies in Epidemiology’ (STROBE) guidelines [33] (See S1 Checklist).

### Procedure

The online survey comprised of the participant information sheet, consent form, and self-report measures described above, using the Qualtrics XM online survey platform. The survey employed forced-response items, with participants given the option to select ‘not applicable’ or ‘prefer not to say’ where appropriate. Participation was voluntary, and no incentive was provided. The survey link was active for a three-week survey window to capture a particular set of circumstances at a point in time in that country. Information was provided about local supports that could be accessed if the self-report questions caused distress.

## Results

### Response rate

A total of 3092 participants consented (Indonesia = 418, Iran = 762, Iraq = 358, Malaysia = 95, Pakistan = 295, Somaliland = 328, Türkiye = 836), of which 2574 (Indonesia = 418, Iran = 499, Iraq = 315, Malaysia = 85, Pakistan = 231, Somaliland = 190, Türkiye = 836) completed the survey, giving the overall completion rate of 83.3%. Most participants completed the survey in local languages, and only 12.5% (n = 323) completed it in English.

### Sociodemographic profile

The age range of participants was between 18–73 years (M = 28.0 years, SD = 10.6), mostly female (64.6%, n = 1663), single (58.0%, n = 1493), and belonging to the Muslim faith (92.2%, n = 2374) (For summary demographics see Table 1, for details see S2 File). Most participants (57.2%, n = 1473) considered their monthly household income average. The majority (81.2%, n = 2789) had at least a tertiary qualification, and some were studying full-time (46.8%, n = 1204) at the time of data collection.

**Table 1:** Showing distribution of demographic characteristics across countries (N = 2574).

	Türkiye	Indonesia	Iraq	Malaysia	Pakistan	Somaliland	Iran	Total
	n (%)	n (%)	n (%)	n (%)	n (%)	n (%)	n (%)	n (%)
***Age*** M (SD)	24.9 (8.6)	35.8 (11.4)	21.5 (6.8)	31.2 (10.3)	22.7 (4.3)	22 (3.5)	35.3 (10.6)	28 (10.6)
***Gender***								
Male	285 (34.1)	145 (34.7)	122 (38.7)	29 (34.1)	43 (18.6)	102 (54.0)	164 (32.9)	890 (34.6)
Female	551 (65.9)	272 (65.1)	191 (60.6)	56 (65.9)	188 (81.4)	85 (45.0)	320 (64.1)	1663 (64.6)
***Religion Islam***	792 (94.7)	370 (88.5)	303 (96.2)	48 (56.2)	231 (100)	190 (100)	440 (88.2)	2374 (92.2)
***Marital status***								
Single	526 (62.9)	118 (28.2)	286 (90.8)	48 (56.5)	194 (84.0)	141 (74.6)	180 (36.1)	1493 (58.0)
In relationship	139 (16.6)	4 (1.0)	10 (3.2)	9 (10.6)	14 (6.1)	15 (7.9)	29 (5.8)	220 (8.6)
Married	158 (18.9)	281 (67.2)	16 (5.1)	26 (30.6)	22 (9.5)	3 (1.6)	270 (54.1)	776 (30.2)
Separated	9 (1.1)	4 (1.0)	1 (0.3)	0 (0.0)	0 (0.0)	30 (15.9)	4 (0.8)	48 (1.9)
Divorced	0 (0.0)	5 (1.2)	1 (0.3)	0 (0.0)	1 (0.4)	0 (0.0)	11 (2.2)	18 (0.7)
Widowed	4 (0.5)	6 (1.4)	1 (0.3)	2 (2.4)	0 (0.0)	0 (0.0)	5 (1.0)	18 (0.7)
***Highest level of** **education***								
No formal education	41 (4.9)	0 (0.0)	51 (16.2)	2 (2.4)	3 (1.3)	10 (5.3)	1 (0.2)	108 (4.2)
Secondary school	125 (15.0)	78 (18.7)	78 (24.8)	4 (4.7)	16 (6.9)	64 (34.0)	10 (2.0)	375 (14.6)
Tertiary qualification	512 (61.2)	16 (3.8)	113 (35.9)	13 (15.3)	7 (3.0)	23 (12.2)	104 (20.8)	788 (30.6)
Bachelor’s degree	126 (15.1)	142 (34.0)	65 (20.6)	46 (54.1)	167 (72.3)	63 (33.5)	218 (43.7)	827 (32.2)
Postgraduate degree	32 (3.8)	182 (43.5)	8 (2.5)	20 (23.5)	38 (16.5)	28 (14.9)	166 (33.3)	474 (18.4)
***Current work or labour force** **status***								
Employed full-time	132 (15.8)	186 (44.5)	18 (5.7)	39 (45.9)	23 (10.0)	16 (8.4)	136 (27.3)	550 (21.4)
Employed part-time	23 (2.8)	28 (6.7)	6 (1.9)	4 (4.7)	7 (3.0)	30 (15.8)	48 (9.6)	146 (5.7)
Studying full-time	533 (63.8)	100 (23.9)	249 (79.0)	34 (40.0)	168 (72.7)	62 (32.6)	58 (11.6)	1204 (46.8)
Unemployed	75 (9.0)	22 (5.3)	23 (7.3)	2 (2.4)	12 (5.2)	8 (4.2)	55 (11.0)	197 (7.7)
***Level of household income***								
Below average	186 (22.2)	50 (12.0)	21 (6.7)	32 (37.6)	22 (9.5)	34 (17.9)	250 (50.1)	595 (23.1)
Average	544 (65.1)	106 (25.4)	249 (79.0)	38 (44.7)	181 (78.4)	133 (70.0)	222 (44.5)	1473 (57.2)
Above average	106 (12.7)	262 (62.7)	45 (14.3)	15 (17.6)	28 (12.1)	23 (12.1)	27 (5.4)	506 (19.7)

*Note: The data is arranged in columns according to the sequence of data collection*.

Sociodemographic characteristics differed across the seven countries. Mean age varied from 21.5 years (SD = 6.8) in Iraq to 35.8 years (SD = 11.4) in Indonesia. The younger population in Iraq is also reflected in higher proportions of full-time students (79.0%, n = 249). Gender distribution showed a notable female predominance in Pakistan (81.4%, n = 188) compared to Somaliland (45.0%, n = 85). Marital status ranged widely, with Indonesia (67.2%, n = 281 married) and Iran (54.1%, n = 270 married) contrasting with Iraq (90.8%, n = 286 single) and Pakistan (84.0%, n = 194 single).

Full-time employment status varied, with Malaysia (45.9%, n = 39) and Indonesia (44.5%, n = 186) showing higher employment than Iraq (5.7%, n = 18) and Somaliland (8.4%, n = 16). Household income was markedly higher in Indonesia (62.7%, n = 262, above average) compared to Iran (50.1%, n = 250, below average). These sociodemographic and temporal differences were accounted for in the subsequent multilevel analyses.

### Trauma exposure

Participants (57.3%, n = 1474) had experienced at least one traumatic event before the COVID-19 pandemic, most commonly a natural disaster (36.1%, n = 928), childhood adversity before the age of 16 years (11.5%, n = 296), and living in a war zone (9.6%, n = 248) (See S2 File). For natural disaster exposure, Indonesia reported the highest prevalence at 58.9% (n = 246), followed closely by Pakistan at 53.2% (n = 123), while Somaliland had the lowest at 17.9% (n = 34), followed by Iraq at 20.6% (n = 65). Exposure to living in a war zone was markedly highest in Iraq at 37.8% (n = 119), with Malaysia reporting the lowest prevalence at 3.5% (n = 3), alongside Pakistan and Türkiye, both at 4.3% (n = 10 and n = 36, respectively). For childhood adversity before age 16, Indonesia exhibited the highest rate at 18.7% (n = 78), followed by Iraq at 17.1% (n = 54), whereas Türkiye and Somaliland reported the lowest rates at 7.4% (n = 62) and 7.9% (n = 15), respectively.

### Pandemic-related variables

Table 2 presents pandemic-related variables across respondents from various countries. Since the COVID-19 pandemic commenced, 56.4% (n = 1451) of participants believed they had contracted the COVID-19 virus, of whom 58.4% (n = 847) tested positive. Additionally, 66.7% (n = 1714) believed a close family member had been diagnosed with COVID-19, of whom 92.5% (n = 1585) tested positive, and 30.2% (n = 517) experienced bereavement. COVID-19 morbidity was notably high in Iran, with 86.3%(n = 429) suspecting infection and 52.6% (n = 261) testing positive, compared to Somaliland (21.3%, n = 40 suspect; 3.7%, n = 7 positive). Family COVID-19 exposure followed similar patterns, with Iraq (81.9%, n = 258 family suspect) and Iran (82.7%, n = 411) reporting higher rates than Indonesia (36.8%, n = 154). At the time of data collection, 16.3% (n = 418) experienced ongoing symptoms, while 26.5% (n = 681) had family members experiencing ongoing symptoms. Additionally, 13.3% of participants (n = 343) were under lockdown or stay-at-home orders at the time of data collection, with the majority (7.3%, n = 261) being from Indonesia.

**Table 2:** Showing Pandemic-related Variables Across Countries (N = 2574).

	Türkiye	Indonesia	Iraq	Malaysia	Pakistan	Somaliland	Iran	Total
	n (%)	n (%)	n (%)	n (%)	n (%)	n (%)	n (%)	n (%)
Lockdown	7 (0.8)	261 (62.4)	25 (7.9)	1 (1.2)	29 (12.6)	13 (6.8)	7 (1.4)	343 (13.3)
Vaccination	767 (91.7)	410 (98.1)	294 (93.3)	85 (100.0)	211 (91.3)	47 (24.7)	451 (90.4)	2265 (88.0)
Intention to get vaccinated	380 (72.5)	392 (93.8)	262 (83.2)	78 (91.8)	156 (67.5)	119 (63.0)	184 (36.9)	1571 (69.5)
***COVID-19 morbidity and mortality***								
COVID-19 suspect	491 (58.9)	167 (40.0)	181 (57.5)	50 (58.8)	93 (40.4)	40 (21.3)	429 (86.3)	1451 (56.5)
COVID-19 positive	246 (29.5)	88 (21.1)	156 (49.5)	37 (43.5)	52 (22.5)	7 (3.7)	261 (52.6)	847 (33.0)
Ongoing COVID-19	88 (10.5)	24 (5.7)	63 (20.0)	29 (34.1)	37 (16.1)	8 (4.2)	169 (34.1)	418 (16.3)
Family COVID-19 suspect	610 (73.1)	154 (36.8)	258 (81.9)	63 (75.0)	137 (59.3)	81 (42.6)	411 (82.7)	1714 (66.7)
Family COVID-19 positive	531 (63.5)	169 (40.5)	247 (78.4)	57 (67.1)	136 (58.9)	65 (34.2)	380 (76.3)	1585 (61.6)
Family Ongoing COVID-19	154 (18.4)	143 (34.2)	83 (26.3)	42 (49.4)	57 (24.7)	22 (11.6)	180 (36.1)	681 (26.5)
Bereavement	197 (23.6)	75 (17.9)	63 (20.0)	6 (7.1)	58 (25.1)	31 (16.3)	91 (18.2)	521 (20.2)
***Financial factors***								
Financial deterioration	499 (59.7)	236 (56.5)	163 (51.7)	53 (62.4)	94 (40.7)	122 (64.2)	259 (51.9)	1426 (55.4)
Job loss	96 (11.5)	49 (11.7)	30 (9.5)	22 (25.9)	43 (18.6)	40 (21.1)	106 (21.2)	386 (15.0)
Essential worker	302 (36.1)	199 (47.6)	147 (46.7)	34 (40.0)	96 (41.6)	87 (45.8)	213 (42.7)	1078 (41.9)
***Source of COVID-19-related information***								
News	595 (71.2)	376 (90.0)	154 (48.9)	67 (78.8)	159 (68.8)	96 (100.0)	280 (56.1)	1727 (69.6)
Facebook/Twitter	384 (45.9)	168 (40.2)	110 (34.9)	59 (69.4)	89 (38.5)	78 (100.0)	173 (34.7)	1061 (43.1)
Health websites	294 (35.2)	214 (51.2)	184 (58.4)	49 (57.6)	51 (22.1)	44 (100.0)	115 (23.0)	951 (39.2)
Word of mouth	149 (17.8)	154 (36.8)	39 (12.4)	33 (38.8)	31 (13.4)	24 (100.0)	86 (17.2)	516 (21.4)
Others	30 (3.6)	74 (17.7)	14 (4.4)	7 (8.2)	16 (6.9)	14 (100.0)	51 (10.2)	206 (8.6)

*Note: The data is arranged in columns according to the sequence of data collection*.

Since the COVID-19 pandemic, most participants (88.0%, n = 2265) received the COVID-19 vaccine, and 69.5% (n = 1571) expressed willingness to get further vaccinated. Interesting trends could be observed in vaccination rates and intention to vaccinate. Türkiye, Pakistan, Malaysia, and Indonesia exhibited high vaccination rates, coupled with strong intentions to vaccinate. In contrast, Somaliland reported a markedly lower vaccination rate (24.7%, n = 47) and intention to vaccinate (63.0%, n = 119). Iran showed a high vaccination rate (90.4%, n = 451) but the lowest intention to vaccinate (36.9%, n = 184), suggesting potential vaccine hesitancy or fatigue. These differences could be influenced by elicitation timing.

A substantial proportion (41.9%, n = 1078) of participants or someone in their household was an essential worker. Financial deterioration affected over half the sample (55.4%, n = 1426), with Somaliland (64.2%, n = 122) and Malaysia (62.4%, n = 53) reporting the highest rates, while Pakistan was lower (40.7%, n = 94). Job loss was most prevalent in Malaysia (25.9%, n = 22) and Iran (21.2%, n = 106) compared to Iraq (9.5%, n = 30).

Information related to COVID-19 was primarily accessed from news sources (67.1%, n = 1727), Facebook/Twitter (41.2%, n = 1061), and health websites (36.9%, n = 951).

### Pandemic-related impacts

The CPIS score range was 5–144 (M = 47.7, SD = 24.0) (See Table 3). The scale correlated moderately with standardized measures of psychological distress (K10_r_s_ = 0.48, 95% CI [0.45, 0.51]; PCL-5_r_s_ = 0.49, 95% CI [0.46, 0.52]) and wellbeing (WHO-5_r_s_ = -0.37, 95% CI [-0.41, -0.34]), reflecting concurrent validity. The precision of the estimates between countries was variable, reflecting the considerable difference in observed sample size.

**Table 3:** Showing the Descriptives on Measures of Psychological Outcomes, Pandemic-related Stress, and Post-traumatic Growth.

		Türkiye (n = 836)	Indonesia (n = 418)	Malaysia (n = 85)	Iraq (n = 315)	Pakistan (n = 231)	Somaliland (n = 190)	Iran (n = 499)	Total (N = 2574)
		n (%)	n (%)	n (%)	n (%)	n (%)	n (%)	n (%)	n (%)
K10	0	479 (57.3)	369 (88.3)	62 (72.9)	190 (60.3)	185 (80.1)	170 (89.5)	290 (58.1)	1745 (67.8)
	1	357 (42.7)	49 (11.7)	23 (27.1)	125 (39.7)	46 (19.9)	20 (10.5)	209 (41.9)	829 (32.2)
WHO-5	0	383 (45.8)	337 (80.6)	47 (55.3)	100 (31.7)	151 (65.4)	160 (84.2)	238 (47.7)	1416 (55.0)
	1	453 (54.2)	81 (19.4)	38 (44.7)	215 (68.3)	80 (34.6)	30 (15.8)	261 (52.3)	1158 (45.0)
PCL5	0	368 (44.0)	373 (89.2)	59 (69.4)	182 (57.8)	166 (71.9)	174 (91.6)	279 (55.9)	1601 (62.2)
	1	468 (56.0)	45 (10.8)	26 (30.6)	133 (42.2)	65 (28.1)	16 (8.4)	220 (44.1)	973 (37.8)
CPIS M (SD)		49.9 (22.4)	42.6 (19.7)	56.5 (28.4)	49.2 (22.3)	42.4 (25.2)	35.9 (22.4)	52.7 (27.1)	47.7 (24.0)
PTGI M (SD)		48.2 (20.9)	70.2 (21.1)	57.2 (21.8)	45.7 (21.9)	51.9 (23.4)	68.2 (23.1)	46.9 (20.4)	53.3 (23.3)

*Note: The data is arranged in columns according to the sequence of data collection. On K10 and PCL5, 0 indicates below the cut-off score and 1 indicates above the cut-off score. On WHO-5, 0 indicates an above cut-off score indicative of wellbeing and 1 indicates a below cut-off score indicative of poorer wellbeing*.

### Psychological outcomes

The K10 (M = 24.6, SD = 10.3), WHO-5 (M = 53.3, SD = 26.8), and PCL-5 (M = 27.3, SD = 20.1) were used to estimate psychological outcomes (See S3 File for country-wise details). The K10 scores showed a strong correlation with PCL5 (r_s_ = 0.81, 95% CI [0.79, 0.82]) and a moderate correlation with WHO-5 (r_s_ = -0.68, 95% CI [-0.70, -0.65]). Additionally, PCL5 demonstrated a moderate correlation (r_s_ = -0.62, 95% CI [-0.65, -0.60]) with WHO-5.

A substantial proportion of participants scored above the cutoff scores on K10 (32.2%, n = 829) and PCL5 (37.8%, n = 973) indicating psychological distress; and below the cutoff score for WHO-5 (45.0%, n = 1158), indicating poor well-being (See Table 3). Iran, Iraq and Türkiye consistently exhibited higher scores on K10 and PCL5 indicating higher psychological distress and scored lower on WHO-5 reflecting poorer wellbeing compared to other countries. Further analysis using superimposed CDF plots and One-way ANOVA will further evaluate these differences.

### Post-traumatic growth

The PTGI scores ranged from 0-105 (M = 53.3, SD = 23.3), showing a spectrum of posttraumatic growth in response to COVID-19 (See Table 3). Indonesia reported the highest PTGI mean score at 70.2 (SD = 21.1), followed closely by Somaliland at 68.3 (SD = 23.1), indicating substantial post-traumatic growth in these populations. In contrast, Iraq had the lowest mean PTGI score at 45.7 (SD = 21.9), followed by Iran at 46.9 (SD = 20.4) and Türkiye at 48.2 (SD = 20.9).

### Factors associated with psychological outcomes

The association of demographic and pandemic-related variables with psychological outcomes was examined using a modified Poisson regression multilevel mixed-effect model [31]. Table 4 shows that gender (female), studying full-time, being unemployed, suspecting COVID-19 (in self or family), COVID-19 vaccine, and financial deterioration due to COVID-19 were related to higher psychological distress. While being in lockdown is associated with lower psychological distress.

**Table 4:** Showing the association of demographic and pandemic-related factors with psychological distress.

	K10	PCL5	WHO-5
	IRR [95% CI]	IRR [95% CI]	IRR [95% CI]
***Demographic variables***			
Gender	**1.49 [1.30, 1.71]**	**1.15 [1.01, 1.30]**	**1.25 [1.10, 1.43]**
Marital status	**0.88 [0.77, 1.00]**	0.97 [0.85, 1.11]	**0.83 [0.72, 0.95]**
Studying fulltime	**1.21 [1.06, 1.38]**	**1.29 [1.13, 1.47]**	**1.15 [1.00, 1.32]**
Unemployed	**1.54 [1.32, 1.80]**	**1.34 [1.10, 1.63]**	**1.31 [1.07, 1.60]**
Previous trauma	1.08 [0.97, 1.21]	1.00 [0.89, 1.12]	1.00 [0.89, 1.12]
***Pandemic-related variables***			
Lockdown at the time of data collection	**0.49 [0.38, 0.64]**	**0.32 [0.24, 0.43]**	**0.56 [0.44, 0.72]**
COVID-19 vaccine	**1.27 [1.05, 1.55]**	**1.46 [1.19, 1.79]**	**1.36 [1.10, 1.67]**
***COVID-19 morbidity and mortality***			
Suspecting COVID-19	**1.28 [1.10, 1.48]**	1.13 [0.98, 1.30]	**1.19 [1.02, 1.38]**
Positive COVID-19 test	1.04 [0.91, 1.20]	1.00 [0.87, 1.16]	1.05 [0.90, 1.22]
Ongoing symptoms of COVID-19	**1.28 [1.10, 1.48]**	1.09 [0.92, 1.29]	1.18 [0.99, 1.40]
Suspecting COVID-19 in the family	**1.24 [1.04, 1.49]**	**1.34 [1.12, 1.60]**	**1.23 [1.03, 1.48]**
Family tested positive for COVID-19	0.96 [0.82, 1.13]	0.97 [0.82, 1.13]	1.06 [0.90, 1.26]
The family has ongoing symptoms of COVID-19	1.02 [0.89, 1.16]	0.92 [0.80, 1.07]	0.96 [0.82, 1.12]
Bereavement	1.03 [0.90, 1.17]	1.10 [0.96, 1.27]	0.91 [0.78, 1.06]
***Financial factors***			
Essential worker	1.00 [0.89, 1.12]	1.00 [0.89, 1.12]	0.95 [0.84, 1.07]
Job loss due to COVID-19	1.11 [0.97, 1.28]	1.04 [0.89, 1.22]	1.03 [0.87, 1.22]
Financial deterioration due to COVID-19	**1.54 [1.36, 1.74]**	**1.28 [1.13, 1.44]**	**1.31 [1.16, 1.49]**

*Note: Items in bold are significant at p < 0.05*.

### Country-wise comparisons

**Fig 1:**
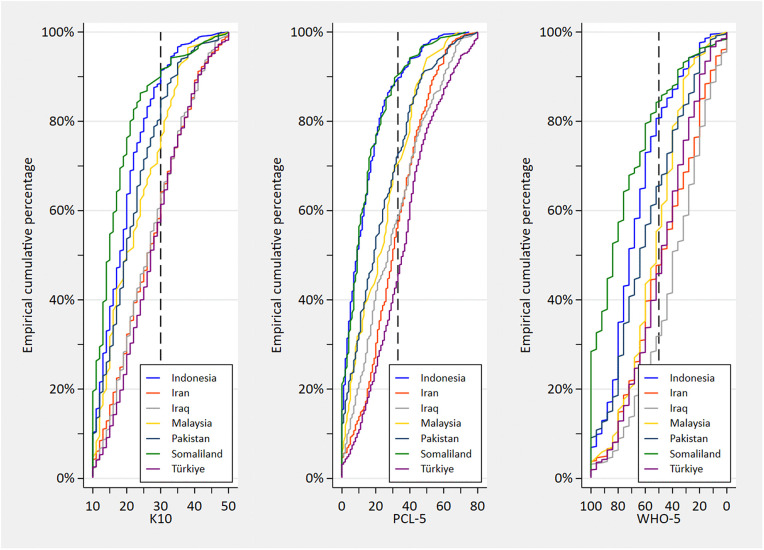
Showing empirical cumulative distributions across countries for psychological outcomes. *Note: A score of 30 and above on K10 is indicative of severe mental distress, a score of 33 and above on PCL-5 is used to screen probable PTSD, and a score of 50 and under on WHO-5 is used to screen clinical depression. The dotted lines in the diagram demonstrate cut-off scores*.

The country-wise comparisons were carried out across psychological outcomes by using the superimposed CDF plots (See Fig 1). The steeper curves on K10 and PCL5 indicate a steeper rate of increase in psychological distress in the cumulative probability for the given data range. In WHO-5, the steeper curve, in contrast, indicates higher wellbeing scores.

One-way ANOVA revealed significant differences in psychological outcome scores across the seven countries. For PCL-5, F(6, 2533)=92.075, p < 0.001, η² = 0.179; for K10, F(6, 2567)=68.785, p < 0.001, η² = 0.139; and for WHO-5, F(6, 2567)=78.337, p < 0.001, η² = 0.155, indicating moderate country effects. Levene’s test showed unequal variances for all outcomes (p < 0.001), so Games-Howell post-hoc tests were conducted (See S4 File). Iran consistently exhibited worse outcomes: higher PCL-5 scores than Türkiye (mean difference = -12.076, p < 0.001, d = -0.93), Somaliland (-23.216, p < 0.001, d = -1.79), and others; higher K10 scores than Türkiye (-7.242, p < 0.001, d = -0.73), Somaliland (-12.811, p < 0.001, d = -1.29), and others; and lower WHO-5 scores than Türkiye (8.454, p < 0.001, d = 0.85), Somaliland (14.818, p < 0.001, d = 1.48), and others. Somaliland often showed better outcomes, particularly versus Iran. These differences highlight the role of pandemic context in psychological outcomes. To control for the potential effects of age and education on differences between countries, an ANCOVA was carried out (See S5 File). The model explained 20.0% variance in PCL-5, 16.4% in K10, and 16.7% in WHO-5. The main effect of the country was significant for psychological outcomes after controlling for covariates.

## Discussion

In this cross-sectional observational study, we examined the psychosocial impacts of the COVID-19 pandemic across seven LMICs. The substantial proportion of participants exhibiting psychological distress echoes global trends reported in meta-analyses [34], highlighting the pervasive nature of mental health challenges during this crisis. Our country-wise comparisons reveal variations in distress levels [35]. For instance, Iran and Türkiye had the steepest distress curve on PCL-5, indicative of a high rate of distress, and this continues to be the case at all levels of distress in response to COVID-19. These findings are consistent with poorer WHO‑5 wellbeing scores observed in the same countries and add to the expanding literature documenting the heterogeneous psychosocial impacts of the pandemic across demographic and geographic settings [36]. It is important to note that prevalence estimates based on K10, PCL‑5, and WHO‑5 cut‑offs represent elevated symptom levels identified through screening tools and do not constitute clinical diagnoses.

The K10, PCL-5, and WHO-5 showed strong to moderate correlations, demonstrating the interconnectedness of various psychological outcomes during the pandemic [37,38]. The CPIS showed excellent internal consistency and moderate correlations with established psychological measures, further demonstrating its validity [39].

The wide variation in PTGI scores aligns with the complex relationship between adversity and post-traumatic growth documented in previous research [40] and the variation in when the survey was conducted. Post-traumatic growth represents adaptive meaning-making and cognitive restructuring in response to adversity [16], and it can coexist with ongoing stress or trauma [6,7], as reflected in our findings. Notably, some countries, such as Indonesia and Somaliland, reported higher levels of post-traumatic growth compared to others. Studies indicate that higher levels of post-traumatic growth may be related to factors such as strong communal ties, collective coping practices, or spiritual and religious frameworks, which could potentially foster resilience and enhance perceptions of personal growth even amid significant stress [41]. It was beyond the scope of this research to evaluate the extent of these factors in the countries surveyed. The inter-country variation in post-traumatic growth we report suggests that systemic responses to crisis situations may influence their impacts. Post-traumatic growth was measured using the PTGI. Other researchers have discussed the underlying nature of post-traumatic growth, positing that it may be used as a coping strategy or reflect positive affective, cognitive, or behavioural change [42]. We did not explore this topic further but accept that perceived growth may reflect positive changes and adaptive coping processes.

The findings highlight the significance of gender, marriage, study and employment status, and ongoing lockdown, extending our understanding of the complex interplay between individual and contextual factors in shaping psychological responses to the pandemic. No model diagnostic issues arose over the course of the analysis, and in some cases, hierarchical models improved model fit as indicated by likelihood ratio statistics. Our findings are consistent with other multinational studies that found an association of depression with younger age, gender (female), and high levels of exposure [5,15,43,44]. Mental health challenges such as depression and anxiety are generally more common among women. We suspect that during highly stressful events like the COVID-19 pandemic, these outcomes can be exacerbated by increased caregiving responsibilities, economic insecurity, and social pressures [45]. The link between marital status and higher distress contrasts with some studies suggesting protective effects of marriage during the pandemic [46], while the associations with demographic and financial factors largely align with previous findings [32].

This study examined the psychosocial impacts of the COVID-19 pandemic across seven LMICs, in cultures and languages where there has been little research. This understanding is vital for developing targeted interventions and support systems to mitigate the psychological impact of future pandemics and similar crises. Unlike many previous studies that focused primarily on mental health disorder prevalence [4,5,12–14], our comprehensive approach, of examining sociodemographic and pandemic-related variables along with standardized psychological and pandemic-related measures, offers a holistic understanding of the psychosocial impacts. This study provides valuable cross-cultural data addressing a critical gap in the literature, which has predominantly focused on high-income countries. The study's multilingual aspect contributes significantly to the translation and availability of mental health measures for cross-cultural comparisons, which can be validated by future studies.

This study has several limitations. The use of non-randomized, online data collection methods, including snowball sampling, although consistent with other multinational studies during the pandemic, introduced potential selection bias and resulted in a non-representative sample. In particular, the sample was skewed toward younger and more highly educated participants, which may limit the generalizability of the findings to broader LMIC populations. Additionally, the sample differed across countries in terms of size, timing of data collection, and demographic composition. Several steps were taken to mitigate these issues. Data were collected within a consistent three-week window in each country to reduce temporal variation. Moreover, cross-site and temporal variations were accounted for using multi-level modelling, with countries included as both random and fixed effects. While these limitations should be considered when interpreting the findings, the methodological controls employed help to ensure the robustness and cross-contextual relevance of the results. Although the participants were given the option to select ‘not applicable’ or ‘prefer not to say,’ the forced-response survey design may have introduced response bias for psychological measures, as participants could feel compelled to answer even if uncertain or uncomfortable. This may affect the prevalence or severity of outcomes, and results should be interpreted with caution. Although this study applied standardized instruments (K10, PCL-5, WHO-5, PTGI) across multiple languages and cultural contexts, formal cross-cultural validation and assessment of measurement invariance were beyond the scope of current paper. it is recommended that they be carried out in future work.

## Conclusion

This study examines the psychosocial impacts of the COVID-19 pandemic across seven LMICs using standardized measures of psychological distress, wellbeing, post-traumatic growth, and pandemic-related distress. This research advances our understanding of the psychological impacts of the COVID-19 pandemic in LMICs, a previously underrepresented area in the literature. Our findings reveal substantial levels of psychological distress across the studied populations, with variations between countries. The identified associations between various demographic, pandemic-related, and socioeconomic factors and psychological outcomes can have implications for policy development, clinical practice, and future research directions. Further studies should examine the trajectory of these psychological impacts across different contexts. Moving forward, mental health considerations must be integrated into pandemic preparedness and response strategies, with particular attention to the unique needs and contexts of LMICs.

## Supporting information

S1 File Measures in english and local language. This file provides the survey measures used in the study in both English and the respective local languages of the participants.
(DOCX)

S1 Checklist STROBE statement for comparing the psychosocial impacts of COVID-19 in seven low- and middle-income countries: a cross-sectional study. (DOCX)

S2 File Distribution of demographic characteristics and trauma exposure (N = 2,574). This file summarizes participants’ demographic data and exposure to trauma across the sample.
(DOCX)

S3 File Descriptive statistics of standardized measures and COVID Psychosocial Impacts Scale (CPIS) across seven LMICs. This file presents descriptive statistics (means, standard deviations, ranges) for standardized psychological measures and the CPIS for participants from the seven low- and middle-income countries included in the study.
(DOCX)

S4 File Pairwise comparisons of psychological outcomes across seven LMICs using games-howell test. This file contains the results of post-hoc Games-Howell tests comparing psychological outcomes between countries.
(DOCX)

S5 File Psychological outcomes by country (Controlling for age and education). This file reports country-level psychological outcomes after adjusting for participants’ age and educational attainment.
(DOCX)

S1 Data Full dataset. (SAV)

## References

[1] World Health Organization. Listings of WHO’s response to COVID-19. 2020. https://www.who.int/news/item/29-06-2020-COVIDtimeline

[2] IHME UW. COVID-19. https://www.healthdata.org/research-analysis/diseases-injuries/COVID

[3] Anderson D, Dominick C, Langley E. The immediate and medium-term social and psycho-social impacts of COVID-19 in New Zealand. Ministry of Social Development New Zealand; 2020.

[4] Chen SX, Ng JCK, Hui BPH, Au AKY, Wu WCH, Lam BCP, et al. Dual impacts of coronavirus anxiety on mental health in 35 societies. Sci Rep. 2021;11(1):8925. doi: 10.1038/s41598-021-87771-1 33903603 PMC8076265

[5] Cénat JM, Noorishad P-G, Kokou-Kpolou CK, Dalexis RD, Hajizadeh S, Guerrier M, et al. Prevalence and correlates of depression during the COVID-19 pandemic and the major role of stigmatization in low- and middle-income countries: a multinational cross-sectional study. Psychiatry Res. 2021;297:113714. doi: 10.1016/j.psychres.2021.113714 33453497 PMC7837092

[6] Beaglehole B, Williman J, Bell C, Stanley J, Jenkins M, Gendall P, et al. Thriving in a pandemic: Determinants of excellent wellbeing among New Zealanders during the 2020 COVID-19 lockdown; a cross-sectional survey. PLoS One. 2022;17(3):e0262745. doi: 10.1371/journal.pone.0262745 35239672 PMC8893611

[7] Jenkins M, Hoek J, Jenkin G, Gendall P, Stanley J, Beaglehole B, et al. Silver linings of the COVID-19 lockdown in New Zealand. PLoS One. 2021;16(4):e0249678. doi: 10.1371/journal.pone.0249678 33793672 PMC8016296

[8] RNZ. COVID global health emergency is over, WHO says. 2023. https://www.rnz.co.nz/news/world/489370/COVID-global-health-emergency-is-over-who-says

[9] Kola L, Kohrt BA, Hanlon C, Naslund JA, Sikander S, Balaji M, et al. COVID-19 mental health impact and responses in low-income and middle-income countries: reimagining global mental health. Lancet Psychiatry. 2021;8(6):535–50. doi: 10.1016/S2215-0366(21)00025-0 33639109 PMC9764935

[10] Hrynick TA, Ripoll S, Carter SE. Broader health impacts of vertical responses to COVID-19 in low- and middle-income countries. The Institute of Development Studies and Partner Organisations; 2020. https://hdl.handle.net/20.500.12413/15626

[11] Rohwerder B. Social impacts and responses related to COVID-19 in low- and middle-income countries. Brighton, UK: Institute of Development Studies; 2020. https://www.ids.ac.uk/publications/social-impacts-and-responses-related-to-COVID-19-in-low-and-middle-income-countries/

[12] Chew NWS, Lee GKH, Tan BYQ, Jing M, Goh Y, Ngiam NJH, et al. A multinational, multicentre study on the psychological outcomes and associated physical symptoms amongst healthcare workers during COVID-19 outbreak. Brain Behav Immun. 2020;88:559–65. doi: 10.1016/j.bbi.2020.04.049 32330593 PMC7172854

[13] Tee M, Wang C, Tee C, Pan R, Reyes PW, Wan X, et al. Impact of the COVID-19 pandemic on physical and mental health in lower and upper middle-income Asian countries: a comparison between the Philippines and China. Front Psychiatry. 2021;11:568929. doi: 10.3389/fpsyt.2020.568929 33633595 PMC7901572

[14] Ammar A, Mueller P, Trabelsi K, Chtourou H, Boukhris O, Masmoudi L, et al. Psychological consequences of COVID-19 home confinement: the ECLB-COVID19 multicenter study. PLoS One. 2020;15(11):e0240204. doi: 10.1371/journal.pone.0240204 33152030 PMC7643949

[15] TMGH-Global COVID-19 Collaborative. Perceived stress of quarantine and isolation during COVID-19 pandemic: a global survey. Front Psychiatry. 2021;12:656664. doi: 10.3389/fpsyt.2021.656664 34113270 PMC8186534

[16] Tedeschi RG, Shakespeare-Finch J, Taku K. Posttraumatic growth: theory, research, and applications. Routledge; 2018. doi: 10.4324/9781315527451

[17] Tanveer S, Schluter PJ, Porter RJ, Boden J, Beaglehole B, Sulaiman-Hill R, et al. Examining the psychosocial impacts of the COVID-19 pandemic: an international cross-sectional study protocol. BMJ Open. 2023;13(4):e067886. doi: 10.1136/bmjopen-2022-067886 37045574 PMC10105919

[18] Bell C, Beaglehole B, Bell R, Tanveer S, Sulaiman-Hill R, Boden J, et al. Learning from previous disasters: Potential pitfalls of epidemiological psychosocial research in the COVID-19 environment. Aust N Z J Psychiatry. 2021;55(7):646–9. doi: 10.1177/0004867421998783 33645256

[19] Sulaiman-Hill RC, Porter R, Tanveer S, Boden J, Beaglehole B, Schluter PJ, et al. Psychosocial impacts on the Christchurch Muslim community following the 15 March terrorist attacks: a mixed-methods study protocol. BMJ Open. 2021;11(10):e055413. doi: 10.1136/bmjopen-2021-055413 34598996 PMC8488282

[20] Tanveer S, Schluter PJ, Beaglehole B, Porter RJ, Boden J, Sulaiman-Hill R, et al. The COVID psychosocial impacts scale: a reliable and valid tool to examine the psychosocial impacts of the COVID-19 pandemic. Int J Environ Res Public Health. 2023;20(11):5990. doi: 10.3390/ijerph20115990 37297593 PMC10252202

[21] Theiner G. A Beginner’s guide to group minds. In: Sprevak M, Kallestrup J, editors. New Waves in Philosophy of Mind. London: Palgrave Macmillan; 2014. 301–22. doi: 10.1057/9781137286734_15

[22] Kessler RC, Barker PR, Colpe LJ, Epstein JF, Gfroerer JC, Hiripi E, et al. Screening for serious mental illness in the general population. Arch Gen Psych. 2003;60(2):184–9. doi: 10.1001/archpsyc.60.2.184 12578436

[23] Andrews G, Slade T. Interpreting scores on the Kessler Psychological Distress Scale (K10). Aust N Z J Public Health. 2001;25(6):494–7. doi: 10.1111/j.1467-842x.2001.tb00310.x 11824981

[24] Topp CW, Østergaard SD, Søndergaard S, Bech P. The WHO-5 well-being index: a systematic review of the literature. Psychother Psychosom. 2015;84(3):167–76. doi: 10.1159/000376585 25831962

[25] Weathers FW, Litz BT, Keane TM. The PTSD Checklist for DSM-5 (PCL-5) – Standard. The National Center for PTSD; 2013. https://www.ptsd.va.gov/

[26] Verhey R, Chibanda D, Gibson L, Brakarsh J, Seedat S. Validation of the posttraumatic stress disorder checklist - 5 (PCL-5) in a primary care population with high HIV prevalence in Zimbabwe. BMC Psychiatry. 2018;18(1):109. doi: 10.1186/s12888-018-1688-9 29685117 PMC5913864

[27] Tedeschi RG, Cann A, Taku K, Senol-Durak E, Calhoun LG. The posttraumatic growth inventory: a revision integrating existential and spiritual change. J Trauma Stress. 2017;30(1):11–8. doi: 10.1002/jts.22155 28099764

[28] Pye V, Taylor N, Clay-Williams R, Braithwaite J. When is enough, enough? Understanding and solving your sample size problems in health services research. BMC Res Notes. 2016;9:90. doi: 10.1186/s13104-016-1893-x 26867928 PMC4751721

[29] IBM Corp. IBM SPSS statistics for windows, Version 28.0. Armonk, NY: IBM Corp. 2021.

[30] StataCorp. Stata statistical software: Release 19. College Station, TX: StataCorp LLC; 2025.

[31] Akoglu H. User’s guide to correlation coefficients. Turk J Emerg Med. 2018;18(3):91–3. doi: 10.1016/j.tjem.2018.08.001 30191186 PMC6107969

[32] Zou G. A modified poisson regression approach to prospective studies with binary data. Am J Epidemiol. 2004;159(7):702–6. doi: 10.1093/aje/kwh090 15033648

[33] von Elm E, Altman DG, Egger M, Pocock SJ, Gøtzsche PC, Vandenbroucke JP, et al. The Strengthening the Reporting of Observational Studies in Epidemiology (STROBE) statement: guidelines for reporting observational studies. Lancet. 2007;370(9596):1453–7. doi: 10.1016/S0140-6736(07)61602-X 18064739

[34] Salari N, Hosseinian-Far A, Jalali R, Vaisi-Raygani A, Rasoulpoor S, Mohammadi M, et al. Prevalence of stress, anxiety, depression among the general population during the COVID-19 pandemic: a systematic review and meta-analysis. Global Health. 2020;16(1):57. doi: 10.1186/s12992-020-00589-w 32631403 PMC7338126

[35] Kowal M, Coll-Martín T, Ikizer G, Rasmussen J, Eichel K, Studzińska A, et al. Who is the most stressed during the COVID-19 pandemic? Data from 26 countries and areas. Appl Psychol Health Well Being. 2020;12(4):946–66. doi: 10.1111/aphw.12234 32996217 PMC7537225

[36] Proto E, Quintana-Domeque C. COVID-19 and mental health deterioration by ethnicity and gender in the UK. PLoS One. 2021;16(1):e0244419. doi: 10.1371/journal.pone.0244419 33406085 PMC7787387

[37] Xiong J, Lipsitz O, Nasri F, Lui LMW, Gill H, Phan L, et al. Impact of COVID-19 pandemic on mental health in the general population: a systematic review. J Affect Disord. 2020;277:55–64. doi: 10.1016/j.jad.2020.08.001 32799105 PMC7413844

[38] Vindegaard N, Benros ME. COVID-19 pandemic and mental health consequences: Systematic review of the current evidence. Brain Behav Immun. 2020;89:531–42. doi: 10.1016/j.bbi.2020.05.048 32485289 PMC7260522

[39] Ransing R, Adiukwu F, Pereira-Sanchez V, Ramalho R, Orsolini L, Teixeira ALS, et al. Mental health interventions during the COVID-19 pandemic: a conceptual framework by early career psychiatrists. Asian J Psychiatr. 2020;51:102085. doi: 10.1016/j.ajp.2020.102085 32413616 PMC7195073

[40] Tedeschi RG, Calhoun LG. Posttraumatic growth: conceptual foundations and empirical evidence. Psychol Inquiry. 2004;15(1):1–18. doi: 10.1207/s15327965pli1501_01

[41] Henson C, Truchot D, Canevello A. What promotes post traumatic growth? A systematic review. Euro J Trauma Dissociat. 2021;5(4):100195. doi: 10.1016/j.ejtd.2020.100195

[42] Jayawickreme E, Infurna FJ. Toward a more credible understanding of post-traumatic growth. J Pers. 2021;89(1):5–8. doi: 10.1111/jopy.12575 32654138

[43] Généreux M, Schluter PJ, Landaverde E, Hung KK, Wong CS, Mok CPY, et al. The evolution in anxiety and depression with the progression of the pandemic in adult populations from eight countries and four continents. Int J Environ Res Public Health. 2021;18(9):4845. doi: 10.3390/ijerph18094845 34062769 PMC8125359

[44] Schluter PJ, Généreux M, Landaverde E, Chan EYY, Hung KKC, Law R, et al. An eight country cross-sectional study of the psychosocial effects of COVID-19 induced quarantine and/or isolation during the pandemic. Sci Rep. 2022;12(1):13175. doi: 10.1038/s41598-022-16254-8 35915133 PMC9341149

[45] Purvis RS, Ayers BL, Rowland B, Moore R, Hallgren E, McElfish PA. “Life is hard”: How the COVID-19 pandemic affected daily stressors of women. Dialogues Health. 2022;1:100018. doi: 10.1016/j.dialog.2022.100018 36776415 PMC9162780

[46] Pieh C, Budimir S, Probst T. The effect of age, gender, income, work, and physical activity on mental health during coronavirus disease (COVID-19) lockdown in Austria. J Psychosom Res. 2020;136:110186. doi: 10.1016/j.jpsychores.2020.110186 32682159 PMC7832650

