# Supplementary material for: Comparing the psychosocial impacts of COVID-19 in seven low- and middle-income countries: A cross-sectional study
Source: PLOS Glob Public Health. 2026 Jun 16;6(6):e0005944. doi: 10.1371/journal.pgph.0005944 (PMC13271434; doi:10.1371/journal.pgph.0005944)
Supplement: S2 File — This file summarizes participants’ demographic data and exposure to trauma across the sample. (DOCX) [file pgph.0005944.s003.docx]

**Supplementary File** **B**

*Distribution of Demographic Characteristics and Trauma Exposure (N=2574)*

|  | Türkiye | Indonesia | Iraq | Malaysia | Pakistan | Somaliland | Iran | Total |
| --- | --- | --- | --- | --- | --- | --- | --- | --- |
|  | n (%) | n (%) | n (%) | n (%) | n (%) | n (%) | n (%) | n (%) |
| ***Age*** M (SD) | 24.9 (8.6) | 35.8 (11.4) | 21.5 (6.8) | 31.2 (10.3) | 22.7 (4.3) | 22 (3.5) | 35.3 (10.6) | 28 (10.6) |
| ***Gender*** |  |  |  |  |  |  |  |  |
| Male | 285 (34.1) | 145 (34.7) | 122 (38.7) | 29 (34.1) | 43 (18.6) | 102 (54.0) | 164 (32.9) | 890 (34.6) |
| Female | 551 (65.9) | 272 (65.1) | 191 (60.6) | 56 (65.9) | 188 (81.4) | 85 (45.0) | 320 (64.1) | 1663 (64.6) |
| Other | 0 (0.0) | 0 (0.0) | 0 (0.0) | 0 (0.0) | 0 (0.0) | 1 (0.5) | 2 (0.4) | 3 (0.1) |
| Prefer not to say | 0 (0.0) | 1 (0.2) | 2 (0.6) | 0 (0.0) | 0 (0.0) | 1 (0.5) | 13 (2.6) | 17 (0.7) |
| ***Religion Islam*** | 792 (94.7) | 370 (88.5) | 303 (96.2) | 48 (56.2) | 231 (100) | 190 (100) | 440 (88.2) | 2374 (92.2) |
| ***Marital status*** |  |  |  |  |  |  |  |  |
| Single | 526 (62.9) | 118 (28.2) | 286 (90.8) | 48 (56.5) | 194 (84.0) | 141 (74.6) | 180 (36.1) | 1493 (58.0) |
| In relationship | 139 (16.6) | 4 (1.0) | 10 (3.2) | 9 (10.6) | 14 (6.1) | 15 (7.9) | 29 (5.8) | 220 (8.6) |
| Married | 158 (18.9) | 281 (67.2) | 16 (5.1) | 26 (30.6) | 22 (9.5) | 3 (1.6) | 270 (54.1) | 776 (30.2) |
| Separated | 9 (1.1) | 4 (1.0) | 1 (0.3) | 0 (0.0) | 0 (0.0) | 30 (15.9) | 4 (0.8) | 48 (1.9) |
| Divorced | 0 (0.0) | 5 (1.2) | 1 (0.3) | 0 (0.0) | 1 (0.4) | 0 (0.0) | 11 (2.2) | 18 (0.7) |
| Widowed | 4 (0.5) | 6 (1.4) | 1 (0.3) | 2 (2.4) | 0 (0.0) | 0 (0.0) | 5 (1.0) | 18 (0.7) |
| ***Highest level of education*** | |  |  |  |  |  |  |  |
| No formal education | 41 (4.9) | 0 (0.0) | 51 (16.2) | 2 (2.4) | 3 (1.3) | 10 (5.3) | 1 (0.2) | 108 (4.2) |
| Secondary school | 125 (15.0) | 78 (18.7) | 78 (24.8) | 4 (4.7) | 16 (6.9) | 64 (34.0) | 10 (2.0) | 375 (14.6) |
| Tertiary qualification | 512 (61.2) | 16 (3.8) | 113 (35.9) | 13 (15.3) | 7 (3.0) | 23 (12.2) | 104 (20.8) | 788 (30.6) |
| Bachelors degree | 126 (15.1) | 142 (34.0) | 65 (20.6) | 46 (54.1) | 167 (72.3) | 63 (33.5) | 218 (43.7) | 827 (32.2) |
| Postgraduate degree | 32 (3.8) | 182 (43.5) | 8 (2.5) | 20 (23.5) | 38 (16.5) | 28 (14.9) | 166 (33.3) | 474 (18.4) |
| ***Current work or labour force status*** | | |  |  |  |  |  |  |
| Employed full-time | 132 (15.8) | 186 (44.5) | 18 (5.7) | 39 (45.9) | 23 (10.0) | 16 (8.4) | 136 (27.3) | 550 (21.4) |
| Employed part-time | 23 (2.8) | 28 (6.7) | 6 (1.9) | 4 (4.7) | 7 (3.0) | 30 (15.8) | 48 (9.6) | 146 (5.7) |
| Studying full-time | 533 (63.8) | 100 (23.9) | 249 (79.0) | 34 (40.0) | 168 (72.7) | 62 (32.6) | 58 (11.6) | 1204 (46.8) |
| Unemployed | 75 (9.0) | 22 (5.3) | 23 (7.3) | 2 (2.4) | 12 (5.2) | 8 (4.2) | 55 (11.0) | 197 (7.7) |
| ***Level of household income*** | | | | | |  |  |  |
| Below average | 186 (22.2) | 50 (12.0) | 21 (6.7) | 32 (37.6) | 22 (9.5) | 34 (17.9) | 250 (50.1) | 595 (23.1) |
| Average | 544 (65.1) | 106 (25.4) | 249 (79.0) | 38 (44.7) | 181 (78.4) | 133 (70.0) | 222 (44.5) | 1473 (57.2) |
| Above average | 106 (12.7) | 262 (62.7) | 45 (14.3) | 15 (17.6) | 28 (12.1) | 23 (12.1) | 27 (5.4) | 506 (19.7) |
|  |  |  |  |  |  |  |  |  |
| ***Trauma exposure before COVID-19*** | | |  |  |  |  |  |  |
| Natural disaster | 303 (36.2) | 246 (58.9) | 65 (20.6) | 23 (27.1) | 123 (53.2) | 34 (17.9) | 134 (26.9) | 928 (36.1) |
| Living in a war zone | 36 (4.3) | 20 (4.8) | 119 (37.8) | 3 (3.5) | 10 (4.3) | 17 (8.9) | 43 (8.6) | 248 (9.6) |
| Childhood adversity before age 16 | 62 (7.4) | 78 (18.7) | 54 (17.1) | 13 (15.3) | 31 (13.4) | 15 (7.9) | 43 (8.6) | 296 (11.5) |
| Physical or sexual assault after 16 | 19 (2.3) | 15 (3.6) | 5 (1.6) | 7 (8.2) | 5 (2.2) | 3 (1.6) | 15 (3.0) | 69 (2.7) |
| Serious physical accident | 43 (5.1) | 31 (7.4) | 17 (5.4) | 9 (10.6) | 11 (4.8) | 17 (8.9) | 41 (8.2) | 169 (6.6) |
| Other | 44 (5.3) | 32 (7.7) | 10 (3.2) | 4 (4.7) | 9 (3.9) | 39 (20.5) | 29 (5.8) | 167 (6.5) |

*Note: The data is arranged in columns according to the sequence of data collection.*
