# Supplementary material for: Comparing the psychosocial impacts of COVID-19 in seven low- and middle-income countries: A cross-sectional study
Source: PLOS Glob Public Health. 2026 Jun 16;6(6):e0005944. doi: 10.1371/journal.pgph.0005944 (PMC13271434; doi:10.1371/journal.pgph.0005944)
Supplement: S3 File — This file presents descriptive statistics (means, standard deviations, ranges) for standardized psychological measures and the CPIS for participants from the seven low- and middle-income countries included in the study. (DOCX) [file pgph.0005944.s004.docx]

**Supplementary File C**

*Descriptive Statistics of Standardized Measures and COVID Psychosocial Impacts Scale (CPIS) across 7 LIMICs*

| **Measures** | | **Türkiye**  **(n=836)** | **Indonesia (n=418)** | **Malaysia**  **(n=85)** | | | **Iraq**  **(n=315)** | **Pakistan (n=231)** | | | **Somaliland (n=190)** | | **Iran**  **(n=499)** | **Total**  **(N=2574)** | | |
| --- | --- | --- | --- | --- | --- | --- | --- | --- | --- | --- | --- | --- | --- | --- | --- | --- |
| **CPIS** | **Score range** | 5-137 | 7-134 | | 10-139 | 6-123 | | | 6-120 | 5-140 | | 7-144 | | | 5-144 |  |
|  | **M (SD)** | 49.9 (22.4) | 42.6 (19.7) | | 56.5 (28.4) | 49.2 (22.3) | | | 42.4 (25.2) | 35.9 (22.4) | | 52.7 (27.1) | | | 47.7 (24) |  |
|  | **σ^2^** | 502.6 | 386.1 | | 806 | 495.8 | | | 636.6 | 500.1 | | 734.9 | | | 574.6 |  |
|  | **SEM** | 0.8 | 1 | | 3.1 | 1.3 | | | 1.7 | 1.6 | | 1.2 | | | 0.5 |  |
| **K10** | **Score range** | 10-50 | 10-50 | | 10-50 | 10-50 | | | 10-48 | 1-50 | | 10-50 | | | 1-50 |  |
|  | **M (SD)** | 27.8 (9.9) | 19.3 (7.7) | | 22.5 (9.5) | 27 (10.2) | | | 21.4 (9) | 17.3 (8.8) | | 27 (10.5) | | | 24.6 (10.3) |  |
|  | **σ^2^** | 98.3 | 59.7 | | 89.6 | 103.6 | | | 80.4 | 77 | | 109.5 | | | 105.8 |  |
|  | **SEM** | 0.3 | 0.4 | | 1 | 0.6 | | | 0.6 | 0.6 | | 0.5 | | | 0.2 |  |
| **PCL-5** | **Score range** | 0-80 | 0-75 | | 0-69 | 0-80 | | | 0-80 | 0-73 | | 0-80 | | | 0-80 |  |
|  | **M (SD)** | 35.6 (20) | 13.3 (14.2) | | 23.6 (17.7) | 29.5 (20.3) | | | 22.5 (18.9) | 13.5 (14.6) | | 30.8 (17.5) | | | 27.3 (20.1) |  |
|  | **σ^2^** | 389.2 | 200.5 | | 313.9 | 414 | | | 357.4 | 213.4 | | 307.6 | | | 403.4 |  |
|  | **SEM** | 0.7 | 0.7 | | 1.9 | 1.2 | | | 1.2 | 1.2 | | 0.8 | | | 0.5 |  |
| **WHO-5** | **Score range** | 0-100 | 0-100 | | 0-100 | 0-100 | | | 0-100 | 0-100 | | 0-100 | | | 0-100 |  |
|  | **M (SD)** | 48.3 (23.5) | 66.9 (21.7) | | 53.7 (21.3) | 39.8 (25.3) | | | 59.8 (25.5) | 75.7 (25.8) | | 47.1 (26.6) | | | 53.3 (26.5) |  |
|  | **σ^2^** | 552.8 | 471 | | 455.4 | 638.8 | | | 649.4 | 665.3 | | 707.3 | | | 700.9 |  |
|  | **SEM** | 0.8 | 1.1 | | 2.3 | 1.4 | | | 1.7 | 1.9 | | 1.2 | | | 0.5 |  |
| **PTGI** | **Score range** | 0-105 | 0-105 | | 0-93 | 0-95 | | | 0-101 | 0-104 | | 0-105 | | | 0-105 |  |
|  | **M (SD)** | 48.2 (20.9) | 70.2 (21.1) | | 57.2 (21.8) | 45.7 (21.9) | | | 51.9 (23.4) | 68.3 (23.1) | | 46.9 (20.4) | | | 53.3 (23.3) |  |
|  | **σ^2^** | 435.3 | 443 | | 473.9 | 477.2 | | | 546.3 | 533.8 | | 416.4 | | | 542.6 |  |
|  | **SEM** | 0.7 | 1 | | 2.4 | 1.2 | | | 1.5 | 1.7 | | 0.9 | | | 0.5 |  |
