# Supplementary material for: Comparing the psychosocial impacts of COVID-19 in seven low- and middle-income countries: A cross-sectional study
Source: PLOS Glob Public Health. 2026 Jun 16;6(6):e0005944. doi: 10.1371/journal.pgph.0005944 (PMC13271434; doi:10.1371/journal.pgph.0005944)
Supplement: S4 File — This file contains the results of post-hoc Games-Howell tests comparing psychological outcomes between countries. (DOCX) [file pgph.0005944.s005.docx]

**Supplementary File D**

*Pairwise Comparisons of Psychological Outcomes Across Seven LMICs Using Games-Howell Test*

|  | | | Mean Difference | *SE* | *p* | 95% Confidence Interval | |
| --- | --- | --- | --- | --- | --- | --- | --- |
|  |  |  |  |  |  | Lower Bound | Upper Bound |
| PCL5 | Indonesia | Iran | -17.49467^*^ | 1.04690 | 0.000 | -20.5882 | -14.4012 |
|  |  | Iraq | -16.13418^*^ | 1.33935 | 0.000 | -20.0982 | -12.1702 |
|  |  | Malaysia | -10.22997^*^ | 2.04253 | 0.000 | -16.3683 | -4.0917 |
|  |  | Pakistan | -9.18353^*^ | 1.42367 | 0.000 | -13.4039 | -4.9631 |
|  |  | Somaliland | -0.18985 | 1.35901 | 1.000 | -4.2270 | 3.8473 |
|  |  | Türkiye | -22.30263^*^ | 0.97774 | 0.000 | -25.1906 | -19.4146 |
|  | Iran | Indonesia | 17.49467^*^ | 1.04690 | 0.000 | 14.4012 | 20.5882 |
|  |  | Iraq | 1.36049 | 1.38946 | 0.958 | -2.7502 | 5.4712 |
|  |  | Malaysia | 7.26469^*^ | 2.07573 | 0.012 | 1.0339 | 13.4955 |
|  |  | Pakistan | 8.31114^*^ | 1.47092 | 0.000 | 3.9532 | 12.6691 |
|  |  | Somaliland | 17.30481^*^ | 1.40842 | 0.000 | 13.1246 | 21.4850 |
|  |  | Türkiye | -4.80796^*^ | 1.04533 | 0.000 | -7.8954 | -1.7205 |
|  | Iraq | Indonesia | 16.13418^*^ | 1.33935 | 0.000 | 12.1702 | 20.0982 |
|  |  | Iran | -1.36049 | 1.38946 | 0.958 | -5.4712 | 2.7502 |
|  |  | Malaysia | 5.90420 | 2.23753 | 0.122 | -0.7836 | 12.5920 |
|  |  | Pakistan | 6.95065^*^ | 1.69158 | 0.001 | 1.9435 | 11.9578 |
|  |  | Somaliland | 15.94432^*^ | 1.63753 | 0.000 | 11.0922 | 20.7964 |
|  |  | Türkiye | -6.16846^*^ | 1.33812 | 0.000 | -10.1282 | -2.2087 |
|  | Malaysia | Indonesia | 10.22997^*^ | 2.04253 | 0.000 | 4.0917 | 16.3683 |
|  |  | Iran | -7.26469^*^ | 2.07573 | 0.012 | -13.4955 | -1.0339 |
|  |  | Iraq | -5.90420 | 2.23753 | 0.122 | -12.5920 | 0.7836 |
|  |  | Pakistan | 1.04645 | 2.28900 | 0.999 | -5.7896 | 7.8825 |
|  |  | Somaliland | 10.04012^*^ | 2.24935 | 0.000 | 3.3154 | 16.7649 |
|  |  | Türkiye | -12.07266^*^ | 2.04173 | 0.000 | -18.2085 | -5.9368 |
|  | Pakistan | Indonesia | 9.18353^*^ | 1.42367 | 0.000 | 4.9631 | 13.4039 |
|  |  | Iran | -8.31114^*^ | 1.47092 | 0.000 | -12.6691 | -3.9532 |
|  |  | Iraq | -6.95065^*^ | 1.69158 | 0.001 | -11.9578 | -1.9435 |
|  |  | Malaysia | -1.04645 | 2.28900 | 0.999 | -7.8825 | 5.7896 |
|  |  | Somaliland | 8.99367^*^ | 1.70719 | 0.000 | 3.9330 | 14.0543 |
|  |  | Türkiye | -13.11910^*^ | 1.42252 | 0.000 | -17.3356 | -8.9027 |
|  | Somaliland | Indonesia | 0.18985 | 1.35901 | 1.000 | -3.8473 | 4.2270 |
|  |  | Iran | -17.30481^*^ | 1.40842 | 0.000 | -21.4850 | -13.1246 |
|  |  | Iraq | -15.94432^*^ | 1.63753 | 0.000 | -20.7964 | -11.0922 |
|  |  | Malaysia | -10.04012^*^ | 2.24935 | 0.000 | -16.7649 | -3.3154 |
|  |  | Pakistan | -8.99367^*^ | 1.70719 | 0.000 | -14.0543 | -3.9330 |
|  |  | Türkiye | -22.11278^*^ | 1.35780 | 0.000 | -26.1458 | -18.0798 |
|  | Türkiye | Indonesia | 22.30263^*^ | 0.97774 | 0.000 | 19.4146 | 25.1906 |
|  |  | Iran | 4.80796^*^ | 1.04533 | 0.000 | 1.7205 | 7.8954 |
|  |  | Iraq | 6.16846^*^ | 1.33812 | 0.000 | 2.2087 | 10.1282 |
|  |  | Malaysia | 12.07266^*^ | 2.04173 | 0.000 | 5.9368 | 18.2085 |
|  |  | Pakistan | 13.11910^*^ | 1.42252 | 0.000 | 8.9027 | 17.3356 |
|  |  | Somaliland | 22.11278^*^ | 1.35780 | 0.000 | 18.0798 | 26.1458 |
| K10 | Indonesia | Iran | -7.679^*^ | 0.602 | 0.000 | -9.46 | -5.90 |
|  |  | Iraq | -7.676^*^ | 0.687 | 0.000 | -9.71 | -5.64 |
|  |  | Malaysia | -3.157 | 1.094 | 0.068 | -6.44 | 0.13 |
|  |  | Pakistan | -2.110^*^ | 0.701 | 0.044 | -4.19 | -0.03 |
|  |  | Somaliland | 2.033 | 0.740 | 0.090 | -0.16 | 4.23 |
|  |  | Türkiye | -8.474^*^ | 0.510 | 0.000 | -9.98 | -6.97 |
|  | Iran | Indonesia | 7.679^*^ | 0.602 | 0.000 | 5.90 | 9.46 |
|  |  | Iraq | 0.002 | 0.741 | 1.000 | -2.19 | 2.19 |
|  |  | Malaysia | 4.521^*^ | 1.129 | 0.002 | 1.14 | 7.90 |
|  |  | Pakistan | 5.569^*^ | 0.753 | 0.000 | 3.34 | 7.80 |
|  |  | Somaliland | 9.712^*^ | 0.790 | 0.000 | 7.37 | 12.05 |
|  |  | Türkiye | -0.795 | 0.580 | 0.818 | -2.51 | 0.92 |
|  | Iraq | Indonesia | 7.676^*^ | 0.687 | 0.000 | 5.64 | 9.71 |
|  |  | Iran | -0.002 | 0.741 | 1.000 | -2.19 | 2.19 |
|  |  | Malaysia | 4.519^*^ | 1.176 | 0.003 | 1.00 | 8.04 |
|  |  | Pakistan | 5.567^*^ | 0.823 | 0.000 | 3.13 | 8.00 |
|  |  | Somaliland | 9.709^*^ | 0.857 | 0.000 | 7.17 | 12.25 |
|  |  | Türkiye | -0.797 | 0.668 | 0.897 | -2.77 | 1.18 |
|  | Malaysia | Indonesia | 3.157 | 1.094 | 0.068 | -0.13 | 6.44 |
|  |  | Iran | -4.521^*^ | 1.129 | 0.002 | -7.90 | -1.14 |
|  |  | Iraq | -4.519^*^ | 1.176 | 0.003 | -8.04 | -1.00 |
|  |  | Pakistan | 1.048 | 1.184 | 0.974 | -2.49 | 4.59 |
|  |  | Somaliland | 5.190^*^ | 1.208 | 0.001 | 1.58 | 8.80 |
|  |  | Türkiye | -5.316^*^ | 1.082 | 0.000 | -8.57 | -2.06 |
|  | Pakistan | Indonesia | 2.110^*^ | 0.701 | 0.044 | 0.03 | 4.19 |
|  |  | Iran | -5.569^*^ | 0.753 | 0.000 | -7.80 | -3.34 |
|  |  | Iraq | -5.567^*^ | 0.823 | 0.000 | -8.00 | -3.13 |
|  |  | Malaysia | -1.048 | 1.184 | 0.974 | -4.59 | 2.49 |
|  |  | Somaliland | 4.143^*^ | 0.868 | 0.000 | 1.57 | 6.71 |
|  |  | Türkiye | -6.364^*^ | 0.682 | 0.000 | -8.39 | -4.34 |
|  | Somaliland | Indonesia | -2.033 | 0.740 | 0.090 | -4.23 | 0.16 |
|  |  | Iran | -9.712^*^ | 0.790 | 0.000 | -12.05 | -7.37 |
|  |  | Iraq | -9.709^*^ | 0.857 | 0.000 | -12.25 | -7.17 |
|  |  | Malaysia | -5.190^*^ | 1.208 | 0.001 | -8.80 | -1.58 |
|  |  | Pakistan | -4.143^*^ | 0.868 | 0.000 | -6.71 | -1.57 |
|  |  | Türkiye | -10.507^*^ | 0.723 | 0.000 | -12.65 | -8.36 |
|  | Türkiye | Indonesia | 8.474^*^ | 0.510 | 0.000 | 6.97 | 9.98 |
|  |  | Iran | 0.795 | 0.580 | 0.818 | -0.92 | 2.51 |
|  |  | Iraq | 0.797 | 0.668 | 0.897 | -1.18 | 2.77 |
|  |  | Malaysia | 5.316^*^ | 1.082 | 0.000 | 2.06 | 8.57 |
|  |  | Pakistan | 6.364^*^ | 0.682 | 0.000 | 4.34 | 8.39 |
|  |  | Somaliland | 10.507^*^ | 0.723 | 0.000 | 8.36 | 12.65 |
| WHO-5 | Indonesia | Iran | 19.809^*^ | 1.595 | 0.000 | 15.10 | 24.52 |
|  |  | Iraq | 27.036^*^ | 1.776 | 0.000 | 21.78 | 32.29 |
|  |  | Malaysia | 13.177^*^ | 2.546 | 0.000 | 5.54 | 20.81 |
|  |  | Pakistan | 7.061^*^ | 1.984 | 0.008 | 1.18 | 12.94 |
|  |  | Somaliland | -8.834^*^ | 2.151 | 0.001 | -15.22 | -2.45 |
|  |  | Türkiye | 18.531^*^ | 1.337 | 0.000 | 14.58 | 22.48 |
|  | Iran | Indonesia | -19.809^*^ | 1.595 | 0.000 | -24.52 | -15.10 |
|  |  | Iraq | 7.227^*^ | 1.856 | 0.002 | 1.74 | 12.72 |
|  |  | Malaysia | -6.632 | 2.603 | 0.151 | -14.43 | 1.16 |
|  |  | Pakistan | -12.747^*^ | 2.056 | 0.000 | -18.84 | -6.66 |
|  |  | Somaliland | -28.643^*^ | 2.218 | 0.000 | -35.22 | -22.07 |
|  |  | Türkiye | -1.278 | 1.442 | 0.975 | -5.54 | 2.98 |
|  | Iraq | Indonesia | -27.036^*^ | 1.776 | 0.000 | -32.29 | -21.78 |
|  |  | Iran | -7.227^*^ | 1.856 | 0.002 | -12.72 | -1.74 |
|  |  | Malaysia | -13.859^*^ | 2.718 | 0.000 | -21.98 | -5.74 |
|  |  | Pakistan | -19.975^*^ | 2.200 | 0.000 | -26.49 | -13.46 |
|  |  | Somaliland | -35.870^*^ | 2.352 | 0.000 | -42.84 | -28.90 |
|  |  | Türkiye | -8.505^*^ | 1.640 | 0.000 | -13.36 | -3.65 |
|  | Malaysia | Indonesia | -13.177^*^ | 2.546 | 0.000 | -20.81 | -5.54 |
|  |  | Iran | 6.632 | 2.603 | 0.151 | -1.16 | 14.43 |
|  |  | Iraq | 13.859^*^ | 2.718 | 0.000 | 5.74 | 21.98 |
|  |  | Pakistan | -6.115 | 2.858 | 0.334 | -14.64 | 2.41 |
|  |  | Somaliland | -22.011^*^ | 2.976 | 0.000 | -30.88 | -13.14 |
|  |  | Türkiye | 5.354 | 2.453 | 0.314 | -2.02 | 12.73 |
|  | Pakistan | Indonesia | -7.061^*^ | 1.984 | 0.008 | -12.94 | -1.18 |
|  |  | Iran | 12.747^*^ | 2.056 | 0.000 | 6.66 | 18.84 |
|  |  | Iraq | 19.975^*^ | 2.200 | 0.000 | 13.46 | 26.49 |
|  |  | Malaysia | 6.115 | 2.858 | 0.334 | -2.41 | 14.64 |
|  |  | Somaliland | -15.896^*^ | 2.513 | 0.000 | -23.34 | -8.45 |
|  |  | Türkiye | 11.470^*^ | 1.863 | 0.000 | 5.94 | 17.00 |
|  | Somaliland | Indonesia | 8.834^*^ | 2.151 | 0.001 | 2.45 | 15.22 |
|  |  | Iran | 28.643^*^ | 2.218 | 0.000 | 22.07 | 35.22 |
|  |  | Iraq | 35.870^*^ | 2.352 | 0.000 | 28.90 | 42.84 |
|  |  | Malaysia | 22.011^*^ | 2.976 | 0.000 | 13.14 | 30.88 |
|  |  | Pakistan | 15.896^*^ | 2.513 | 0.000 | 8.45 | 23.34 |
|  |  | Türkiye | 27.366^*^ | 2.040 | 0.000 | 21.30 | 33.43 |
|  | Türkiye | Indonesia | -18.531^*^ | 1.337 | 0.000 | -22.48 | -14.58 |
|  |  | Iran | 1.278 | 1.442 | 0.975 | -2.98 | 5.54 |
|  |  | Iraq | 8.505^*^ | 1.640 | 0.000 | 3.65 | 13.36 |
|  |  | Malaysia | -5.354 | 2.453 | 0.314 | -12.73 | 2.02 |
|  |  | Pakistan | -11.470^*^ | 1.863 | 0.000 | -17.00 | -5.94 |
|  |  | Somaliland | -27.366^*^ | 2.040 | 0.000 | -33.43 | -21.30 |
| *Note.* The mean difference is significant at the 0.05 level. | | | | | | | |
