# Supplementary material for: Comparing the psychosocial impacts of COVID-19 in seven low- and middle-income countries: A cross-sectional study
Source: PLOS Glob Public Health. 2026 Jun 16;6(6):e0005944. doi: 10.1371/journal.pgph.0005944 (PMC13271434; doi:10.1371/journal.pgph.0005944)
Supplement: S5 File — This file reports country-level psychological outcomes after adjusting for participants’ age and educational attainment. (DOCX) [file pgph.0005944.s006.docx]

**Supplementary File E**

*Psychological Outcomes by Country (Controlling for Age and Education)*

| **Outcome** | ***Effect*** | ***df*** | ***F*** | ***p*** |
| --- | --- | --- | --- | --- |
| **K10** | **Country** | 8, 2562 | 62.82 | < .001 |
|  | **Age** | 1, 2562 | 49.58 | < .001 |
|  | **Education** | 1, 2562 | 15.14 | < .001 |
| **WHO-5** | **Country** | 8, 2562 | 64.37 | < .001 |
|  | **Age** | 1, 2562 | 26.42 | < .001 |
|  | **Education** | 1, 2562 | 7.75 | .005 |
| **PCL-5** | **Country** | 8, 2529 | 78.95 | < .001 |
|  | **Age** | 1, 2529 | 49.42 | < .001 |
|  | **Education** | 1, 2529 | 7.00 | .008 |

*Notes.* All effects (country, age, education) were statistically significant (p < .05) across outcomes, indicating that country differences in psychological outcomes persist after controlling for age and education.
